# Integrating dietary supplementation with poppy (*Papaver somniferum* L.) seed meal: effects on growth performance, nutrient digestibility, and faecal microbiota in broilers

**DOI:** 10.5194/aab-67-73-2024

**Published:** 2024-02-15

**Authors:** Riaz Khan, Muhammad Tahir, Shabana Naz, Ibrahim A. Alhidary, Shamsuddin Shamsi, Sifa Dai, Rifat Ullah Khan, Vincenzo Tufarelli

**Affiliations:** 1 Department of Animal Nutrition, Faculty of Animal Husbandry and Veterinary Sciences, The University of Agriculture Peshawar, Peshawar, Pakistan; 2 Department of Zoology, Government College University, Faisalabad, Pakistan; 3 Department of Animal Production, College of Food and Agriculture, King Saud University, Riyadh, Saudi Arabia; 4 College of Veterinary Sciences, Faculty of Animal Husbandry and Veterinary Sciences, The University of Agriculture Peshawar, Peshawar, Pakistan; 5 Department of Pharmaceutical and Life, Jiujiang Bozheng Institute of Biotechnology Industry Sciences, Jiujiang University, No. 551, Qianjindong Road, Jiujiang, 332005, Jingxi Province, PR China; 6 Department of Precision and Regenerative Medicine and Jonian Area (DiMePRe-J), Section of Veterinary Science and Animal Production, University of Bari “Aldo Moro”, s.p. Casamassima km 3, 70010 Valenzano, Italy

## Abstract

The study investigated the effects of supplementing broiler diets with poppy (*Papaver somniferum* L.) seed meal (PSM) on growth performance, nutrient digestibility, faecal microbiota, and blood profiles. A total of 250 unsexed 1 d old broilers were allocated to five distinct treatment groups. PSM was incorporated into the diets at varying levels: 0 % (PSM ${}_{0}$ ), 5 % (PSM ${}_{5}$ ), 10 % (PSM ${}_{10}$ ), 15 % (PSM ${}_{15}$ ), and 20 % (PSM ${}_{20}$ ). The findings indicated that growth performance, including weight gain, feed intake, and feed conversion ratio (FCR), was significantly improved ( P<0.05 ) in the PSM ${}_{20}$ group compared to the control. However, carcass weight experienced a notable decrease ( P<0.05 ) in the PSM ${}_{20}$ group. Regarding nutrient digestibility, PSM supplementation led to reduced crude protein digestibility. Nevertheless, apparent metabolizable energy and ash content were significantly enhanced ( P<0.05 ) in the PSM ${}_{15}$ and PSM ${}_{20}$ groups. Notably, faecal microbiota also experienced substantial improvement ( P<0.05 ) in the PSM ${}_{20}$ group. In conclusion, the study demonstrates that incorporating poppy seed meal at a rate of 20 % in broiler diets enhances growth performance, improves nutrient digestibility, and positively influences faecal microbiota composition.

## 1 Introduction

The worldwide proliferation of animal production has led to a notable surge in the demand for animal feed proteins. In the contemporary context, proteins derived from plants have taken precedence as the primary source of protein in animal diets (Patsios et al., 2020; Imtiaz et al., 2023). Remarkably, it has been documented that soybean meal (SBM) contributes as much as 70 % of these plant-based proteins (Kim et al., 2019). Regrettably, the reliability of SBM as a protein source has been compromised due to its escalating price resulting from the intense demand. This phenomenon can be attributed to significant competition between human food and animal feed consumption. As a result, there is a growing imperative to seek out alternative sources of protein that are both cost-effective and rich in nutritional content. This circumstance has prompted nutritionists to take a keen interest in the utilization of locally cultivated protein sources.

The poppy plant (*Papaver somniferum*) serves as a natural reservoir of opium. While its origins can be traced back to Asia Minor, it is presently cultivated in regions across the globe that share similar climates. Opium contains a variety of active components, with morphine and codeine standing out as its primary constituents (McKim, 1997; Akinci and Bayram, 2003). The components of the poppy plant that are utilized include dried raw fruits, scratched raw fruits, leaves, seeds, as well as the oil extracted from the seeds and the pulp that remains after oil extraction (Bayram et al., 2008). Studies have indicated that poppy seeds possess elevated levels of crude proteins, crude fibre, and crude energy, accompanied by a substantial quantity of essential minerals (Özcan and Atalay, 2006). Additionally, poppy seed meal (PSM) falls under the category of oilseed meals that are abundant in polyunsaturated fatty acids (Azcan et al., 2004). The fatty acid composition of PSM demonstrates a more favourable profile compared to rapeseed oil, and its fatty acid distribution closely mirrors that of sunflower seed oil (Eklund and Ågren, 1975).

A prior investigation focused on laying hens and revealed that incorporating varying proportions of PSM (7.5 % and 15 %) into their diets led to favourable outcomes in terms of feed consumption and egg weight (Küçükersan et al., 2009). The research suggested the viability of substituting PSM for SBM given its higher protein content, cost-effectiveness, and wide availability in various global regions. Furthermore, there have been instances of positive impacts resulting from the inclusion of PSM in animal diets. For instance, the supplementation of PSM in the diet was found to enhance growth and egg production in quails (Akinci and Bayram, 2003). Likewise, laying hens that were provided with a diet containing an additional 2.5 % of poppy seed oil (PSO) exhibited heightened egg production and improved egg quality (Bayram et al., 2008). Notably, significant improvements were observed in the growth performance of broiler chickens that were fed a diet containing 10 %–25 % PSM, as highlighted by Bayram et al. (2006). Nevertheless, the available evidence regarding the potential impact of poppy seed supplementation on growing–finishing broilers remains quite limited. Consequently, the objective of this study was to assess the response of growing–finishing broilers to a diet supplemented with PSM. The assessment will encompass aspects such as growth performance, nutrient digestibility, and composition of the faecal microbiota.

## 2 Materials and methods

The white poppy seed (*Papaver somniferum* L.) meal was obtained from the local market in Peshawar. An analysis of its nutrient composition, as outlined in Table 1, was conducted, and the meal was subsequently incorporated into the experimental diets based on its analysed composition.

**Table 1:** Analysed composition of poppy seed meal.

Nutrient	Composition
Dry matter	91 %
Moisture	9.0 %
Crude protein	29 %–32 %
Metabolizable energy	2500 kcal kg ${}^{-1}$
Ether extract	24 %
Crude fibre	10 %
Ash content	6.5 %

### 2.1 Birds, housing, and diets

A total of 250 unsexed, 1 d old broilers were distributed into five distinct treatment groups. Each treatment group was comprised of three replicates, with each replicate containing 15 birds housed in separate pens. Over the course of the study, all the birds were provided unrestricted access to both commercial feed (as detailed in Table 1) and water. The broilers were accommodated within metallic cages that measured 80 × 60 × 45 cm, equipped with feeders and water drinkers for their convenience.

The lighting regimen initially provided 14 h of illumination per day, which was subsequently extended to 16 h. The dietary regimen comprised corn–soybean-based mash feeds, formulated in accordance with the guidelines set forth by the National Research Council in 1994 (as depicted in Table 1). The birds were administered vaccinations by a veterinarian at appropriate intervals, with distilled water used as the medium. Ambient temperatures were documented on a daily basis, fluctuating between 14 and 23 ${}^{\circ }$ C as the highest and lowest recorded temperatures. Relative humidity levels remained within the range of 60 % to 70 %. Throughout the study, the broilers were reared in wire cages, ensuring consistent management, hygienic conditions, and a stable environment. The experimental diets were formulated using identical basal components. To create variation, PSM was added to the diets in different proportions: 0 % (PSM ${}_{0}$ ), 5 % (PSM ${}_{5}$ ), 10 % (PSM ${}_{10}$ ), 15 % (PSM ${}_{15}$ ), and 20 % (PSM ${}_{20}$ ). These adjustments were applied to both the control group and the experimental groups. The formulations of the diets utilized in the experiments are detailed in Table 2.

**Table 2:** Composition and calculated analysis of the experimental diets ${}^{1}$ .

Ingredients (% unless noted)	PSM ${}_{0}$	PSM ${}_{5}$	PSM ${}_{10}$	PSM ${}_{15}$	PSM ${}_{20}$
Ground yellow corn	54.77	53.80	52.23	50.71	49.21
Soybean meal (dehulled)	33.68	29.06	25.10	20.87	16.94
Poppy seed meal	0.00	5.00	10.00	15.00	20.00
Corn gluten meal (60 %)	1.25	1.50	2.20	2.60	3.00
Canola meal	0.75	1.20	1.25	1.50	1.50
Fish meal	2.25	2.50	2.50	3.00	3.30
Vegetable oil	3.00	3.00	3.00	3.00	3.00
Molasses	1.00	1.00	1.00	1.00	1.00
Limestone	1.30	1.02	0.76	0.47	0.19
Dicalcium phosphate	1.31	1.23	1.19	1.07	0.99
Common salt	0.43	0.43	0.44	0.44	0.44
DL-methionine	0.14	0.14	0.11	0.10	0.12
L-lysine HCL	0.00	0.00	0.10	0.12	0.20
Vit : min premix ${}^{2}$	0.12	0.12	0.12	0.12	0.12
Total	100	100	100	100	100
Calculated composition					
CP, %	23.32	23.00	23.00	23.00	23.00
ME, kcal g ${}^{-1}$	3.07	3.07	3.07	3.07	3.07
Calcium, %	1.00	1.00	1.00	1.00	1.00
npP – non-phytate P, %	0.45	0.45	0.45	0.45	0.45
Lysine (%)	1.28	1.22	1.24	1.21	1.22
Methionine (%)	0.58	0.54	0.53	0.54	0.58
Methionine + cystine (%)	0.90	0.91	0.91	0.92	0.96
Threonine (%)	0.88	0.86	0.85	0.84	0.83
Tryptophan (%)	0.30	0.29	0.27	0.26	0.25

${}^{1}$ PSM: poppy seed meal. ${}^{2}$ PSM ${}_{0}$ : basal diet without PSM. PSM ${}_{5}$ : basal diet with 5 % PSM supplementation. PSM ${}_{10}$ : basal diet with 10 % PSM supplementation. PSM ${}_{15}$ : basal diet with 15 % PSM supplementation. PSM ${}_{20}$ : basal diet with 20 % PSM supplementation.

#### 2.1.1 Performance traits

At the outset of the experiment, the initial average body weight of the chicks was documented. Subsequently, the weekly averages for both body weight and feed intake (FI) were recorded during the first, second, and third weeks of the experiment. For the first week, the average body weight gain (BWG) was computed by subtracting the average body weight of chicks at the end of the first week from their initial body weight at the start of the first week. Similarly, the second week's WG was determined by deducting the average body weight observed during the first week from the average body weight noted at the conclusion of the second week. The third week's BWG was calculated using the same method as applied in the second week. The FI for each week was determined by subtracting the amount of feed refused from the total quantity of feed provided. The feed conversion ratio (FCR) was derived by dividing the unit weight gain by the unit feed intake. Upon reaching day 28, the average body weight of the chicks and the remaining feed within each replicate were weighed. Subsequently, 2 chicks were randomly chosen from each replicate, totalling 40 chicks, and were then euthanized. The birds were dissected, and measurements were taken for the weights of the carcass and liver.

#### 2.1.2 Nutrient digestibility

In order to explore nutrient digestibility, three birds from each replicate were isolated 3 d prior to the conclusion of the study. As a tracer, chromic oxide (Cr ${}_{2}$ O ${}_{3}$ ) was incorporated into the experimental diets at a rate of 0.3 %. Subsequently, the faecal matter from each replicate was gathered on consecutive days and preserved at a temperature of -40 ${}^{\circ }$ C until the time of analysis. The diets and faeces samples were subjected for the dry matter (DM) contents at 105 ${}^{\circ }$ C using the oven-drying method. Gross energy and nitrogen (N) content of feed and faeces samples were measured using the bomb calorimeter (Leco, 500) and Kjeldahl method, respectively, at the Centre of Animal Nutrition, Veterinary Research Institute, Directorate of Livestock and Development, Peshawar.

The nutrient digestibility or retention and the apparent metabolizable energy (AME) value were determined by Hong et al. (2002) as

1digestibility/retention(%)=100-[100×(dietCr2O3/faecalCr2O3)×(faecalnutrient/dietnutrient)],2AME(kcalkgDM-1)=dietGE-(faecalGE×dietCr2O3/faecalCr2O3),

where GE is gross energy.

#### 2.1.3 Faecal microbiota

Fresh faecal samples were directly obtained from two birds per replication on the final day (day 28) of the experiment to analyse faecal microbial counts. One gram of faecal sample was then thoroughly mixed with 9 mL of 1 % peptone broth. For the assessment of total viable bacterial counts in the faecal samples, 10-fold serial dilutions were applied and plated onto MacConkey agar plates and *Lactobacilli* medium III agar plates, facilitating the isolation of *Escherichia coli* and *Lactobacillus*, respectively. *Lactobacilli* medium III agar plates underwent incubation in an anaerobic environment at 39 ${}^{\circ }$ C for 48 h, while MacConkey agar plates were incubated at 37 ${}^{\circ }$ C for 24 h. The colonies of *E. coli* and *Lactobacillus* were promptly enumerated upon plate removal from the incubator. The microflora concentration was subsequently expressed as log ${}_{10}$ CFU (colony-forming units) per gram of faeces.

#### 2.1.4 Statistical analysis

All the analyses were conducted using SPSS for Windows (version 14.0, 2020, SPSS Chicago, IL, 2011). To ascertain significant distinctions among the treatments, the Tukey test was employed, with a significance level set at 5 %.

## 3 Results

The data concerning the average weight gain, feed intake, and feed efficiency of chicks fed various levels of PSM during the first week of the experiment are presented in Table 3. Notably, no significant differences ( P>0.05 ) were observed in terms of body weight gain, feed intake, and FCR among the control and experimental groups.

**Table 3:** Average weight gain, feed intake, and feed efficiency in broiler chicks fed different levels of PSM-based diets.

Diets ${}^{*}$	Weight gain	Feed intake	FCR
Week 1			
PSM ${}_{0}$	191.25 ± 12.50	327.50 ± 17.56	1.72 ± 0.12
PSM ${}_{5}$	208.75 ± 11.09	280.00 ± 18.71	1.35 ± 0.14
PSM ${}_{10}$	205.50 ± 8.23	337.50 ± 10.41	1.65 ± 0.10
PSM ${}_{15}$	206.25 ± 7.50	332.50 ± 20.21	1.61 ± 0.08
PSM ${}_{20}$	207.50 ± 13.23	313.75 ± 22.87	1.52 ± 0.19
Week 2			
PSM ${}_{0}$	370.00 ${}^{b}$ ± 17.80	543.75 ${}^{\mathrm{ab}}$ ± 5.06	1.48 ${}^{\mathrm{ab}}$ ± 0.08
PSM ${}_{5}$	362.50 ${}^{b}$ ± 17.08	541.25 ${}^{b}$ ± 17.50	1.50 ${}^{\mathrm{ab}}$ ± 0.12
PSM ${}_{10}$	358.25 ${}^{b}$ ± 13.50	536.25 ${}^{b}$ ± 7.50	1.50 ${}^{\mathrm{ab}}$ ± 0.04
PSM ${}_{15}$	353.20 ${}^{b}$ ± 18.56	549.88 ${}^{\mathrm{ab}}$ ± 17.94	1.56 ${}^{a}$ ± 0.05
PSM ${}_{20}$	401.25 ${}^{a}$ ± 17.02	563.75 ${}^{a}$ ± 10.31	1.41 ${}^{b}$ ± 0.04
Week 3			
PSM ${}_{0}$	491.25 ${}^{b}$ ± 16.52	837.50 ± 2.89	1.71 ${}^{a}$ ± 0.06
PSM ${}_{5}$	492.50 ${}^{b}$ ± 16.58	833.13 ± 2.39	1.70 ${}^{a}$ ± 0.06
PSM ${}_{10}$	506.25 ${}^{\mathrm{ab}}$ ± 15.48	832.50 ± 3.19	1.65 ${}^{\mathrm{ab}}$ ± 0.06
PSM ${}_{15}$	525.97 ${}^{a}$ ± 8.93	833.97 ± 3.52	1.59 ${}^{b}$ ± 0.03
PSM ${}_{20}$	506.81 ${}^{\mathrm{ab}}$ ± 7.58	832.03 ± 5.28	1.64 ${}^{\mathrm{ab}}$ ± 0.03
Week 4			
PSM ${}_{0}$	1052.50 ${}^{c}$ ± 10.41	1708.75 ${}^{a}$ ± 17.76	1.62 ${}^{a}$ ± 0.03
PSM ${}_{5}$	1063.75 ${}^{\mathrm{bc}}$ ± 17.97	1654.38 ${}^{b}$ ± 14.77	1.56 ${}^{\mathrm{bc}}$ ± 0.03
PSM ${}_{10}$	1070.00 ${}^{\mathrm{bc}}$ ± 17.32	1706.25 ${}^{a}$ ± 15.54	1.60 ${}^{\mathrm{ab}}$ ± 0.04
PSM ${}_{15}$	1085.42 ${}^{b}$ ± 17.50	1716.35 ${}^{a}$ ± 29.11	1.58 ${}^{\mathrm{abc}}$ ± 0.03
PSM ${}_{20}$	1115.56 ${}^{a}$ ± 24.29	1709.53 ${}^{a}$ ± 30.28	1.53 ${}^{c}$ ± 0.04

Means in each column with the same letter are not significantly different at a P level of 0.05. ${}^{*}$ PSM ${}_{0}$ : basal diet without PSM. PSM ${}_{5}$ : basal diet with 5 % PSM supplementation. PSM ${}_{10}$ : basal diet with 10 % PSM supplementation. PSM ${}_{15}$ : basal diet with 15 % PSM supplementation. PSM ${}_{20}$ : basal diet with 20 % PSM supplementation.

The impact of feeding different levels of PSM had a significant effect on the weight gain ( P<0.05 ), feed intake ( P<0.07 ), and feed efficiency ( P=0.10 ) of chicks during the second week. The enhancement in body weight gain was notably significant only when the diet included 20 % PSM. Interestingly, the inclusion of PSM up to 15 % did not result in an improved weight gain during the second week of the experiment. However, diets containing 5 % and 10 % PSM seemed to lead to a reduction in feed intake when compared to the PSM ${}_{0}$ diet. The feed intake demonstrated an increase beyond 10 % PSM in the diet, reaching its peak ( P<0.05 ) when the diet included 20 % PSM, in contrast to the diets with 5 % and 10 % PSM. In terms of FCR, there were significant differences between chicks fed various levels of PSM and those fed a diet without PSM. However, within the PSM-based diets, chicks consuming a diet with 15 % PSM exhibited poorer FCR compared to those on the PSM ${}_{20}$ diet. Notably, FCR did not improve until the diet contained 20 % PSM.

In the third week, there was an observable trend of weight gain improvement in chicks on a PSM ${}_{10}$ diet. When the PSM inclusion was elevated to 15 % in the diet, a remarkable weight gain of 525.97 g was achieved in the chicks ( P<0.05 ). However, further increasing the PSM content to 20 % did not result in improved weight gain compared to PSM ${}_{15}$ . Notably, the impact of feeding PSM-based diets on feed intake in chicks did not exhibit significance. Regarding FCR, there was a significant reduction ( P<0.05 ) in FCR for PSM ${}_{15}$ when compared to PSM ${}_{0}$ , indicating improved efficiency.

During the fourth week, there was a noteworthy increase in weight gain, with PSM ${}_{20}$ exhibiting significantly higher weight gain ( P<0.05 ) in comparison to PSM ${}_{0}$ . Conversely, feed intake witnessed a significant decrease ( P<0.05 ) in PSM ${}_{5}$ compared to the other treatments. Additionally, a lower FCR was observed in PSM ${}_{20}$ when compared to PSM ${}_{0}$ , indicating improved feed efficiency.

The impact of PSM supplementation on total live weight, carcass weight, and liver weight can be found in Table 4. Total live weight and carcass weight demonstrated a significant increase ( P<0.05 ) in PSM ${}_{20}$ when compared to PSM ${}_{10}$ and PSM ${}_{15}$ . However, there was no significant alteration ( P>0.05 ) in liver weight between the control and treatment groups.

**Table 4:** The total live weight, carcass weight, and liver weight of broiler chicks fed different levels of PSM-based diets.

Diets ${}^{*}$	Total live weight	Carcass weight	Liver weight
PSM ${}_{0}$	1192.50 ${}^{c}$ ± 10.41	823.00 ${}^{\mathrm{ab}}$ ± 29.83	34.75 ± 1.71
PSM ${}_{5}$	1203.75 ${}^{c}$ ± 17.97	859.00 ${}^{a}$ ± 29.34	34.75 ± 1.50
PSM ${}_{10}$	1210.00 ${}^{\mathrm{bc}}$ ± 17.32	794.25 ${}^{\mathrm{bc}}$ ± 21.70	34.20 ± 1.63
PSM ${}_{15}$	1225.42 ${}^{b}$ ± 17.50	787.00 ${}^{\mathrm{bc}}$ ± 18.78	34.10 ± 1.41
PSM ${}_{20}$	1255.56 ${}^{a}$ ± 24.29	778.00 ${}^{c}$ ± 23.85	33.50 ± 1.29

Means in each column with the same letter are not significantly different at a P level of 0.05. ${}^{*}$ PSM ${}_{0}$ : basal diet without PSM. PSM ${}_{5}$ : basal diet with 5 % PSM supplementation. PSM ${}_{10}$ : basal diet with 10 % PSM supplementation. PSM ${}_{15}$ : basal diet with 15 % PSM supplementation. PSM ${}_{20}$ : basal diet with 20 % PSM supplementation.

**Table 5:** The digestibility of crude protein (CP), ash, organic matter (OM), and apparent metabolizable energy (AME) values in broiler chicks fed different levels of PSM-based diets.

Diets ${}^{*}$	CP	AME	Ash	OM
	(%)	(kcal kg ${}^{-1}$ )	(%)	(%)
PSM ${}_{0}$	64.07 ${}^{a}$ ± 0.96	3092.39 ${}^{d}$ ± 36.87	25.19 ${}^{b}$ ± 2.99	76.48 ± 0.75
PSM ${}_{5}$	60.83 ${}^{b}$ ± 1.12	3187.68 ${}^{c}$ ± 38.34	25.47 ${}^{b}$ ± 2.53	76.76 ± 0.75
PSM ${}_{10}$	59.66 ${}^{b}$ ± 1.09	3213.39 ${}^{\mathrm{bc}}$ ± 32.59	26.76 ${}^{b}$ ± 1.90	77.08 ± 0.70
PSM ${}_{15}$	57.01 ${}^{c}$ ± 0.77	3244.16 ${}^{\mathrm{ab}}$ ± 32.85	35.16 ${}^{a}$ ± 1.46	77.39 ± 0.55
PSM ${}_{20}$	55.05 ${}^{d}$ ± 0.93	3288.24 ${}^{a}$ ± 20.90	37.19 ${}^{a}$ ± 1.88	76.92 ± 0.32

Means in each column with the same letter are not significantly different at a P level of 0.05. ${}^{*}$ PSM ${}_{0}$ : basal diet without PSM. PSM ${}_{5}$ : basal diet with 5 % PSM supplementation. PSM ${}_{10}$ : basal diet with 10 % PSM supplementation. PSM ${}_{15}$ : basal diet with 15 % PSM supplementation. PSM ${}_{20}$ : basal diet with 20 % PSM supplementation.

The digestibility of crude protein (CP), ash, organic matter (OM), and apparent metabolizable energy (AME) values in broiler chicks fed varying levels of PSM-based diets is provided in Table 5. The findings revealed that PSM ${}_{15}$ and PSM ${}_{20}$ led to a decrease ( P<0.05 ) in CP digestibility compared to the control. However, AME and ash values displayed significant increases ( P<0.05 ) in PSM ${}_{20}$ when compared to PSM ${}_{0}$ . No significant difference was observed in the content of OM.

**Table 6:** Effects of dietary supplementation of poppy seed incorporation on faecal microbiota in broilers in the finisher phase (log ${}_{10}$ CFU g ${}^{-1}$ ).

Group	*Lactobacillus*	*E. coli*
PSM ${}_{0}$	7.40 ${}^{c}$	4.56 ${}^{a}$
PSM ${}_{5}$	8.12 ${}^{b}$	4.45 ${}^{a}$
PSM ${}_{10}$	8.14 ${}^{b}$	4.39 ${}^{a}$
PSM ${}_{15}$	8.23 ${}^{b}$	4.21 ${}^{b}$
PSM ${}_{20}$	8.50 ${}^{a}$	4.11 ${}^{a}$
SEM (standard error of the mean)	0.17	0.03
P value	0.04	0.04

Mean values with a column with different superscripts differ significantly ( P<0.05 ).

Table 6 presents the impact of varying PSM levels on the faecal microbiota (*Lactobacillus* and *E. coli*) of both the control and treatment groups. The findings indicate a significant increase ( P<0.05 ) in the level of *Lactobacillus* with PSM ${}_{20}$ and a significant decrease ( P<0.05 ) in the level of *E. coli* compared to the control.

## 4 Discussion

The utilization of PSM as a feed supplement in contemporary animal nutrition has gained attention in recent decades. Feed supplements are frequently employed to enhance the general growth, health, and well-being of animals. Poppy seed meal, recognized for its high protein content (Statham, 1984), is a well-regarded option in this regard. Nevertheless, despite its ongoing expansion in global production, there have been mounting apprehensions linked to the trade of poppy seeds. These concerns could potentially stem from international regulations and controls governing the trade of poppy seeds (UN, 1999). In the present study, significantly higher growth performance was found in PSM ${}_{20}$ compared to the control. Previously, Bayram et al. (2006) reported a significant increase in broiler body weight gain with a 25 % inclusion of PSM in the diet. However, research regarding the performance of pigs fed PSM-supplemented diets remains quite limited. Moreover, inconsistent results are observed in the literature due to variations in PSM dosage, diverse animal models, and discrepancies in dietary compositions. As an example, a study conducted by Bayram and Akinci (1998) on growing Japanese quails revealed that the inclusion of 20 % PSM in the diet had no noticeable impact on growth performance. Similarly, Yıldız et al. (2006) found no impact on body weight, live-weight gain, and feed consumption when substituting 20 % of SBM with PSM. Similarly, Akyildiz (1984) reported that inclusion of PSM at levels ranging from 8 % to 16 % in broiler diets did not result in noticeable variations in daily weight gain and feed intake, even when substituting 50 % to 100 % of conventional soybean meal (CSM). However, specific instances have arisen where the addition of PSM (up to 15 % in the diet) along with yeast culture (*Saccharomyces cerevisiae*) at a rate of 0.10 % demonstrated positive effects on egg weight and eggshell quality parameters (such as eggshell thickness) without adversely affecting egg production and feed efficiency, as observed in the study by Küçükersan et al. (2009). Feed intake was superior in PSM ${}_{20}$ , which can contribute to an increase in diet flavour. Similar results were obtained by Küçükersan et al. (2009) in laying hens.

Interestingly, the carcass weight in PSM ${}_{20}$ decreased significantly compared to PSM ${}_{5}$ . The observed decrease in carcass weight despite improved weight gain in the PSM ${}_{20}$ group compared to the PSM ${}_{5}$ group may be attributed to several factors such as anti-nutritional factors and the metabolism of nutrients, especially energy and challenges in digesting and utilizing certain nutrients effectively at higher levels of PSM. This could lead to variations in the deposition of muscle mass and, consequently, carcass weight. The reduction in carcass weight observed in the PSM ${}_{20}$ group, despite improved weight gain, may have several potential implications for overall carcass quality and market suitability such as meat-to-bone ratio, market value, and consumer preferences.

In the current study, digestibility of CP deceased with increasing levels of PSM. However, AME and ash production increased with increasing PSM levels. Overall, there is a dearth of information regarding the nutrient digestibility of PSM. Muhizi and Kim (2021) noted that the digestibility of protein remained unaffected when pigs were fed a PSM diet. The precise mechanism through which PSM decreases CP digestibility and consequently leads to reduced carcass weight remains unclear. However, the Eklund and Ågren (1975) and Statham (1984) observations were made of poor nitrogen levels in infants fed on poppy milk. Poppy seeds and their derivatives are notably deficient in lysine and tryptophan. To mitigate the potential deficiency in chicks, synthetic lysine supplementation was employed when PSM was included at levels exceeding 15 % in the diet. The reduction in CP digestibility and the hindered carcass weight could suggest the presence of either anti-nutrients within PSM or inadequate availability of crucial amino acids from PSM. It is possible that some unidentified anti-nutrients in PSM might have interfered with the biological functions of these essential amino acids, thereby contributing to the observed decrease in growth performance.

Interestingly, an intriguing observation was made regarding the increasing inclusion of PSM, which revealed a gradual rise in AME values – a trend that contrasted with the trend observed in CP digestibility. Specifically, AME values for PSM ${}_{10}$ and PSM ${}_{15}$ were measured at 121 and 151.8 kcal kg ${}^{-1}$ higher than PSM ${}_{0}$ , respectively. The AME value reached its peak at 195.9 kcal kg ${}^{-1}$ , with the highest value observed in chicks fed PSM ${}_{20}$ compared to those on PSM ${}_{0}$ . This pattern of response was similarly observed in ash retention.

Previous studies on poultry have demonstrated that diets formulated with imbalanced and/or high energy-to-protein ratios result in elevated carcass fat pads in broiler chicks. Although the data regarding abdominal fat pads were not recorded in this study, it was evident that more abdominal fat pads were observed in chicks fed PSM-based diets at the time of slaughtering (data not shown). This led to the assumption that the higher AME levels could potentially contribute to the accumulation of abdominal fat and subsequently lead to reduced carcass weight (Muhizi and Kim, 2021).

In broiler diets, the protein content is typically maintained at a higher level than the metabolizable energy during the starter phase, which is then comparatively reduced during the finisher phase. Deviating from these ratios in any feeding phase could adversely impact optimal performance (Smith et al., 1998). This study illustrates that, with increased PSM inclusion, the energy that should ideally be allocated for protein deposition in the body actually contributes to lipogenesis, leading to the accumulation of abdominal fat and subsequently resulting in lower carcass weight (Neto et al., 2000; Aletor et al., 2000; Hai and Blaha, 2000). Additionally, the experimental diets based on PSM exhibited a progressive increase in gross energy content as the inclusion of PSM increased, as determined through laboratory analysis. Notably, the diet containing 20 % PSM displayed 140 kcal kg ${}^{-1}$ more energy than PSM ${}_{0}$ . This finding implies that the AME of this specific PSM variety was greater than what has been reported in the existing literature. This widening ratio of CP to energy could potentially play a role in affecting CP digestibility and contributing to the reduced carcass weight observed in broiler chicks.

In the current study, *Lactobacillus* species increased and *E. coli* decreased significantly with increasing levels of PSM. In the study of Muhizi and Kim (2021), no significant difference was found between the control and experimental groups fed a 2 % PSM diet in terms of *E. coli* and *Lactobacillus* species in faecal microbiota of growing pigs. In support of our study, Caridi (2002) documented that various species of *Lactobacillus* exhibit inhibitory effects on *E. coli*. Consequently, an elevation in *Lactobacillus* count might result in a reduction in *E. coli* count due to competition for nutrient utilization or the production of lactic acid within the digestive system. The observed improvement in faecal microbiota in the PSM ${}_{20}$ group may be attributed to several potential mechanisms. PSM could act as a pre-biotic, fostering the growth of beneficial gut bacteria like *Lactobacillus*. The diverse microbial community influenced by PSM may enhance digestive function and nutrient utilization. Potential anti-inflammatory effects and the fibre content in PSM could contribute to a healthier gut environment, while modulation of gut pH and metabolite production from PSM breakdown might positively influence microbial populations. Additionally, interactions between PSM components and the gut mucosa could impact bacterial attachment, collectively promoting more robust and balanced gut microbiota in broilers.

Our study's practical applications for broiler production involve determining optimal PSM inclusion levels, balancing nutrient profiles for growth and carcass quality, evaluating economic feasibility, and considering industry adoption. Insights into market positioning and future research directions contribute to informed poultry nutrition practices. These findings offer actionable guidance for industry stakeholders, aiding in the formulation of cost-effective, performance-optimized broiler diets that align with consumer preferences and market demands.

## 5 Conclusion

From the results of the present study, it was concluded that poppy seed meal at a rate of 20 % enhanced growth performance, heightened nutrient digestibility, and improved faecal microbiota in broilers.

## Data Availability

The data presented in this study are available within the article (see Sect. 3).
